# How Many Instruments Do We Need? Strengths, Weaknesses, and Potentials for Further Developments of Digital Health Literacy Measurement Instruments: Narrative Overview of Reviews and Content Analysis

**DOI:** 10.2196/91603

**Published:** 2026-08-28

**Authors:** Pauline Kaboth, Lorenz Harst, Jochen Schmitt, Madlen Scheibe

**Affiliations:** 1 Center for Evidence-Based Healthcare, Faculty of Medicine and University Hospital Carl Gustav Carus, TUD Dresden University of Technology, Fetscherstraße 74, Dresden, 01307, Germany, 49 371 333 35323

**Keywords:** electronic health literacy, eHealth literacy, digital health, telemedicine, review, qualitative research, digital health literacy

## Abstract

**Background:**

With the ongoing digitalization of health care, digital health literacy (DHL) is becoming increasingly important, requiring appropriate measurement instruments (DHLMIs). However, the wide range of available DHLMIs makes selection difficult and raises questions about their suitability and comparability.

**Objective:**

This review aimed to provide an overview of available DHLMIs for adult populations and to compare their dimensions, identifying overlaps and differences to determine which (key) dimensions are most commonly studied and thus define DHL.

**Methods:**

A narrative overview of reviews was conducted. The database search was conducted in November 2024, with an updated search in March 2026, in PubMed and Google Scholar using specific search terms and predefined inclusion and exclusion criteria. This was complemented by a forward citation search in Web of Science. All identified records were screened in a multistage process. At the review level, systematic and scoping reviews published since January 1, 2020, were included that analyzed DHLMIs in adult populations. At the primary study level, studies were included in which DHLMIs were used for self-assessment, performance-based evaluation, or a combination of both. Data extraction was performed by one reviewer and verified by a second reviewer. Data on underlying theories, methods of data collection (performance-based or self-reported), and target groups were extracted. DHL dimensions, their definitions, and the associated items were categorized by 2 researchers using qualitative content analysis according to Kuckartz.

**Results:**

A total of 12 reviews were included. Of the 170 measurement instruments examined in these reviews, 33 (19%) were selected for detailed analysis after applying the inclusion and exclusion criteria. The majority of the included measurement instruments (n=20, 61%) were not based on a theory, 20 (61%) measured DHL exclusively via self-report, and 21 (64%) addressed specific target groups. The measurement instruments encompassed a total of 209 original dimensions of DHL. The number of dimensions measured per instrument varied between 2 and 22. The qualitative content analysis identified a total of 30 assigned dimensions. Key dimensions captured in almost all instruments, although under different names, include evaluating health information, using health information, researching health information, and the ability to use technology.

**Conclusions:**

The large number of measurement instruments and original dimensions makes it difficult to select suitable instruments for measuring DHL and to compare and synthesize study results. Nevertheless, we were able to identify key dimensions as relevant regardless of target groups and types of digital applications, which could be used to develop a core outcome set for DHL. Our findings offer a foundation for refining existing instruments and developing new ones. They provide practical guidance for researchers and health care professionals in selecting suitable DHLMIs. Additionally, our review underlines the importance of theory to ensure content validity and comparability of DHLMIs.

## Introduction

### Background

Health literacy is a key resource for achieving and maintaining good health [1,2]. It is defined as the knowledge, motivation, and ability of individuals to access, understand, evaluate, and apply health information to make informed decisions that support or improve health and quality of life [3]. While health information was traditionally accessed through print media, (audio-)visual broadcasts, or direct interaction with health care professionals, due to increasing digitalization, the internet has become the dominant source of health-related information [4,5].

To enable effective use of this information, individuals need to have digital health literacy (DHL). Understanding how this concept has developed over time is essential for contextualizing the diversity of available measurement instruments and the dimensions they cover. The concept of eHealth literacy, on which DHL is based, was first defined by Norman and Skinner in 2006 as the ability to seek, find, understand, and appraise health information from electronic sources and apply the knowledge gained to address or solve a health problem [6,7]. Their Lily Model described eHealth literacy as a set of 6 core literacies: traditional literacy, health literacy, information literacy, scientific literacy, media literacy, and computer literacy [7]. As digital health technologies evolved beyond static web resources to include interactive, mobile, and social media platforms, the concept of “digital health literacy” was introduced to capture the broader and more complex skill set required to effectively engage with these new environments [8]. Norman [9] himself acknowledged these limitations in his 2011 update, and subsequent scholars have proposed revised or entirely new definitions and models (eg, [10,11]). This progress has prompted the development of broader conceptual frameworks. The conceptual heterogeneity of the field is illustrated by a concept analysis by Ban et al [8], which identified multiple competing definitions and traced how the concept has continued to evolve in response to technological and contextual change.

The diversity of available theoretical models, coupled with the absence of a universally accepted definition, is reflected in the large number of measurement instruments available [10-12]. These differ in their underlying theoretical frameworks, target populations, assessment methods—ranging from performance-based to self-reported approaches—and the dimensions they cover. The wide range of instruments available makes selection difficult and raises questions about the suitability and comparability of study results. This challenge is not limited to DHL but is also present in clinical settings, for example, in the assessment of disease severity using instruments for atopic dermatitis [13].

Previous reviews have analyzed the psychometric properties of available digital health measurement instruments (DHLMIs) using the COSMIN methodology [12,14] or conducted content analyses restricted to instrument development studies for the general population [15] or focused exclusively on either self-reported [12] or performance-based instruments [16]. However, no review has yet applied a structured qualitative content analysis to systematically examine and synthesize the dimensional landscape across the full breadth of DHLMIs in active research use, including instruments for both the general population and specific target groups, such as older adults or individuals with chronic conditions. This review aimed to address this gap.

### Study Objectives

The objective of our study was to provide a comprehensive overview of existing DHLMIs, both self-reported and performance-based, to systematically analyze and synthesize the dimensions across instruments, including those developed for the general population and those targeting specific populations, such as older adults or individuals with chronic conditions. We focused on the underlying theory, assessed dimensions, assessment methods (ie, performance-based, self-report, or mixed method approaches), and target populations. Rather than aiming to identify a single universal instrument, our goal was to identify overlaps and differences between instruments and to determine which key dimensions are most commonly operationalized across DHLMIs, thereby contributing to a more systematic understanding of how digital health literacy is currently conceptualized and measured in research practice. This analysis is intended to support researchers in making more informed decisions about which instrument to use, to provide instrument developers with an evidence-based starting point, and to contribute to the development of a core outcome set for DHL.

## Methods

### Overview

We conducted a narrative overview of reviews, including their primary studies, to provide a meta-level synthesis concerning the aforementioned research aim. We opted for a narrative review because it offers a more flexible structure and is not as strictly bound to a search and evaluation protocol. This allows us to more easily integrate different perspectives, theories, and study types into this study and provide a broader overview of the research field [17]. This approach is particularly suitable for heterogeneous topics where existing research is diverse, making it easier to present and discuss the variety of approaches and findings [18].

Another reason for choosing a narrative review is that it supports our primary objective. Our primary objective was to analyze the dimensions represented in the measurement instruments. This question is inherently qualitative in nature and is best addressed through an interpretive synthesis. Despite our narrative approach, we have applied several systematic principles in key areas of our work, which are described in more detail below.

### Search Strategy

We conducted an electronic database search on November 4, 2024, with an additional update search on March 26, 2026. Searches were performed in PubMed and Google Scholar to capture relevant interdisciplinary sources. Furthermore, a forward citation search in Web of Science was conducted as part of the update search to enhance the comprehensiveness of the literature search.

Our search terms included the free text terms: “Digital Health Literacy,” “eHealth Literacy,” “Digital Literacy,” “Digital Health Competence,” “Health Information Literacy,” “Digital Health Skills,” “Digital Health Knowledge,” “Online Health Literacy,” and “Electronic Health Literacy,” in various combinations with the terms “tool” or “instrument” via the Boolean operator “AND.”

### Study Selection

**Figure 1.**
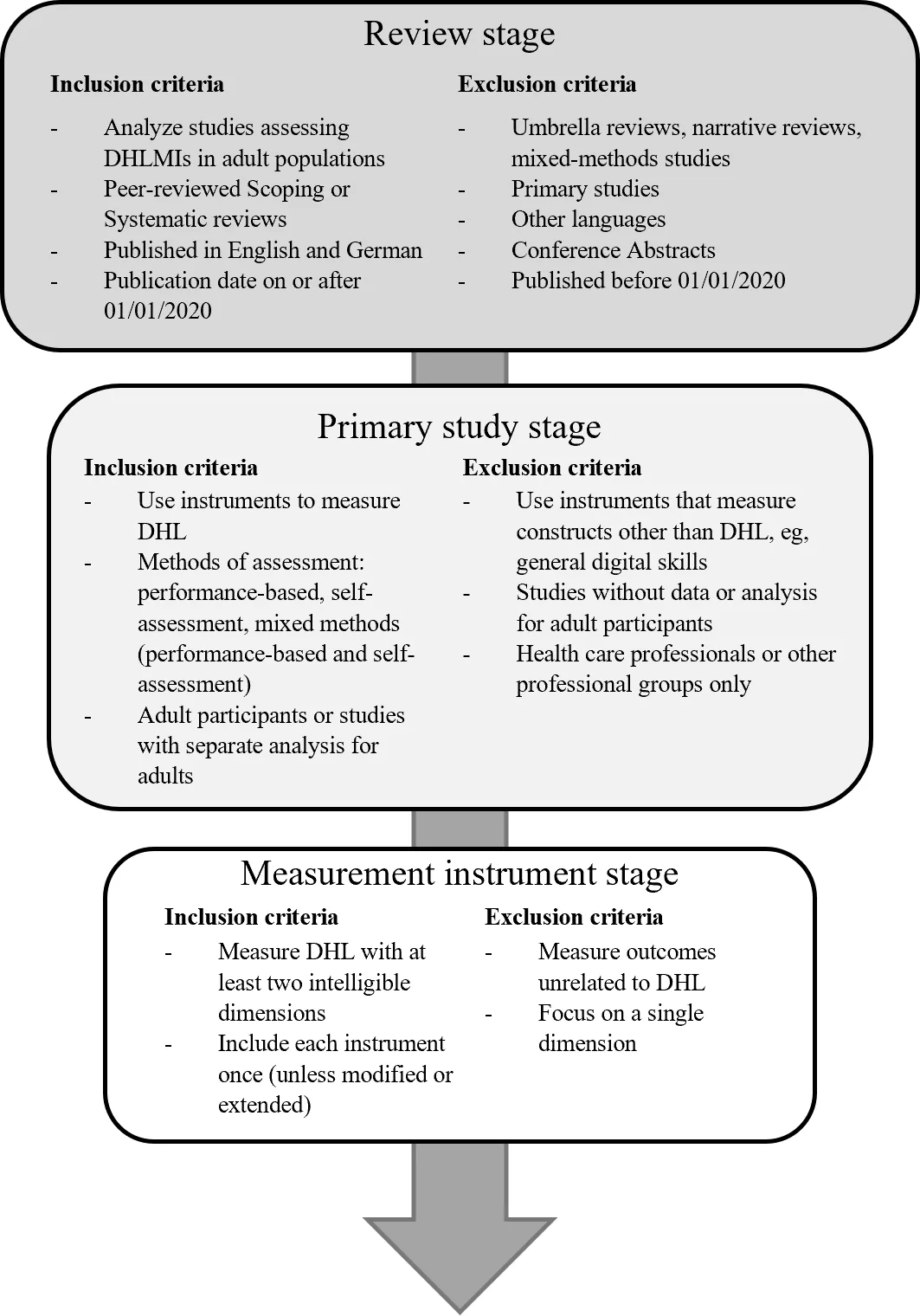
Inclusion and exclusion criteria. DHL: digital health literacy; DHLMI: digital health measurement instruments.

A 3-stage inclusion process at the review, primary study, and measurement instrument level was used to arrive at the number of instruments relevant to our analysis (Figure 1).

At the review stage, we included reviews that analyzed studies assessing DHLMIs in adult populations. Specifically, we considered systematic and scoping reviews published in English or German since January 1, 2020, to present the current evidence. In addition, the search periods of the reviews also cover primary studies from earlier years, which, in turn, examine measurement instruments developed at that time. We excluded umbrella reviews, mixed methods studies, narrative reviews, primary studies, and research published in languages other than English or German. Conference abstracts and studies published before January 1, 2020, were also excluded. To focus on the most recent evidence on the topic, we also excluded conference abstracts and studies published before January 1, 2020. At the primary study stage, we included studies that used instruments to measure DHL through self-report methods, performance-based assessments, or a combination of both. We restricted inclusion to studies conducted with adult participants or studies that included mixed age groups if a separate analysis for adult participants was available. Studies conducted exclusively with health care professionals or other professional groups were excluded, as this review focuses on DHLMIs designed to assess personal DHL in the context of individual health management, rather than professional digital health competence applied in clinical practice.

At the instrument stage, we included instruments published in English or German that measured DHL by considering at least two intelligible dimensions. DHL is conceptualized as an inherently multidimensional construct across established theoretical frameworks, including the Lily Model [7], the eHealth Literacy Framework [19], and the Transactional Model of eHealth Literacy [20]. Instruments capturing only a single dimension fail to adequately operationalize this complexity. Therefore, we restricted inclusion to instruments measuring at least two distinct dimensions of DHL. Additionally, we excluded instruments that measured only health literacy without digital attributes or digital literacy without health-related components.

If one measurement instrument was used in more than one study, we only included the instrument once in our analyses, unless the original measurement instrument was supplemented or modified in another study. If a measurement instrument was used in multiple studies, we selected the most recent primary study to ensure that all previous developments and refinements of the instrument were taken into account.

### Data Extraction

We developed a standardized data extraction sheet within the research team. All relevant information on the included reviews, primary studies, and measurement instruments—including dimensions, theory, methods of assessment (performance-based or self-assessment), target groups, contexts of use, and purpose—was extracted and recorded in said extraction sheet. Data extraction was carried out by one reviewer (PK) and verified for accuracy and completeness by a second reviewer (LH).

### Data Synthesis

To determine which conceptual dimensions (*original dimensions*) were covered by the included measurement instruments, we conducted a structured qualitative content analysis following the approach by Kuckartz [21]. Therefore, we reviewed the dimension definitions and items from each instrument and grouped them into broader categories, which we called *assigned dimensions*. Where dimensions were not explicitly described, the assignment to our categories was based directly on the items. These categories were developed and refined through joint coding by 2 researchers (PK and LH). Differences in coding were discussed and resolved by consensus within the entire research team (PK, LH, and MS). Dimensions for which no clear definitions or corresponding items were available or accessible were excluded from this analysis. Subsequently, the assigned dimensions were systematically grouped into overarching domains.

To assess the prevalence of each assigned dimension, we applied two counts of frequency: (1) frequency across instruments, where a dimension was counted once per instrument regardless of how often it appeared within it; and (2) frequency per original dimension, where each occurrence of the assigned dimension across different original dimensions within the same instrument was counted separately. The latter step was necessary as we found different definitions for the measurement instruments for the assigned dimensions. This dual approach enabled us to capture both the overall representation and the specific distribution of each assigned dimension.

## Results

### Overview of Included Studies Focusing on DHLMIs

We identified 12 reviews [4,11,12,14-16,22-27] that met the inclusion criteria and were included in our analysis (Table 1).

These reviews were published between 2021 and 2026 and included a total of 589 primary studies. Five (42%) of the included reviews were systematic reviews [4,12,14,24,27], and the remaining reviews were scoping reviews. Seven (58%) reviews examined DHLMIs for specific target groups or settings such as adults living in community environments [4], people in hospital settings [23], older adults [22,24,26,27], or patients of health and social care professionals [25], while the other reviews studied DHL as a generic construct [11,12,14-16]. Four (33%) reviews [4,11,12,14] considered only self-reported measurements, and one (8%) review [16] examined only performance-based measurements. Six (50%) of the 12 reviews investigated both self-report and performance-based measurements [15,23-27]. The median number of primary studies included was 40 (range 2-251). The median number of included instruments was 10, with a range of 2 to 44. The primary studies included in the reviews comprised a total of 170 instruments. After removing the duplicates, a total of 94 instruments remained. Of these 94 instruments, 51 measured DHL, of which 33 met our inclusion and exclusion criteria.

**Table 1.** Characteristics of included reviews.

Author, year	Study design	Inclusion period (as reported)	Aim of the study regarding DHL^a^ instruments	Performance-based or self-reported	Included studies, n	Participants in all included primary studies, N	Included instruments, n	Included instruments measuring DHL, n	Target group or setting of the review
Ahn and Kim, 2024 [4]	Systematic review	NR^b^	Evaluate the characteristics and psychometric properties of questionnaires designed to measure DHL	S^c^	21	11,325	21	19	Adolescents and adults living in community settings
Bai et al, 2025 [22]	Scoping review	2014 and March 2024	Identify tools for assessing digital health literacy to guide dietetic practice in screening patients for digital care needs and training	S	66	40,517	8	8	Middle-aged and older adults (age >45 y) in clinical, community, or population settings
Crocker et al, 2023 [16]	Scoping review	Up to June 2021	Identify tools measuring DHL based on objective performance	P^d^	33	8404	33	29	No specific target group
Délétroz et al, 2026 [14]	Systematic review	January 2000 to 2024	Evaluate measurement properties of patient-reported outcome measures of eHealth literacy	S	89 studies and 3 reports	NR	17	15	Adult population (age >18 y)
Dijkman et al, 2023 [23]	Scoping review	1991‐2022	Provide an overview of tools for measuring (digital) health literacy in hospitals	P and S	251	NR	44	7	Hospital setting
Faux-Nightingale et al, 2022 [11]	Scoping review	Up to February 25, 2021	Assess available tools that can be used to evaluate DHL	S	56	NR	5	5	No specific target group/setting
Huang et al, 2023 [24]	Systematic review	Up to January 13, 2021	Examine the diagnostic accuracy of DHL tools	P and S	2	365	2	2	Older adults (age >60 y)
Kaihlanen et al, 2023 [25]	Scoping review	Up to February 2023	Identify available evaluation tools for assessing the client’s potential and suitability for using digital health	P and S	19	NR	12	9	Patients of health and social care professionals
Lee et al, 2021 [12]	Systematic review	Up to March 3, 2021	Identify the currently available instruments for measuring eHealth literacy and to evaluate their measurement properties	S	57	NR	7	7	No specific target group or setting
Wang and Luan, 2022 [26]	Scoping review	NR	Identify available evaluation tools to assess DHL	P and S	47	NR	5	5	Older adults (age ≥65 y)
Wang et al, 2025 [15]	Scoping review	2006 to June 2024	Evaluate the characteristics, effectiveness, and limitations of existing eHealth literacy assessment instruments	P and S	13	NR	13	13	No specific target group
Xie et al, 2022 [27]	Systematic review	NR	Identify, appraise, and synthesize research evidence of the association between DHL and health outcomes	P and S	24	11,778	3	3	Older adults (age >60 y)

a DHL: digital health literacy.

b NR: not reported in the reviews.

c S: self-reported.

d P: performance based.

### Overview of DHLMIs

In total, 33 different DHLMIs were identified that met our inclusion criteria (Table 2). These were used 234 times in the primary studies (Table 3). The measurement instruments reviewed by Délétroz et al [14] and Wang et al [15,26] are not included in this count, as none of these reviews reported information on the frequency of use of instruments in primary studies. The most frequently used instruments used in the primary studies included in the reviews were the 8-item eHealth Literacy Scale [6] (n=163, 72%), the eHealth Literacy Questionnaire [28] (n=13, 6%), the Digital Health Literacy Instrument Screening Tool [29] (n=12, 5%), the eHealth Literacy Assessment toolkit [30] (n=8, 3%), the Transactional eHealth Literacy Instrument [20] (n=5, 2%), the Electronic-Health Literacy Scale [31] (n=3, 1%), and the Extended eHealth Literacy Scale [32] (n=3, 1%). Three instruments (Digital Health Technology Literacy Assessment Questionnaire [33], eHEALS+not specified [34], and Readiness and Enablement Index for Health Technology [35]) were used twice. All other 23 instruments were used in only one primary study [29,36-57].

These were developed between 2006 and 2023. Thirteen (40%) of these were developed in North America [6,20,30,31,37,38,40-42,44,50,53,56], 10 (30%) were developed in Asia [33,34,36,39,43,45-47,51,57], and 10 (30%) were developed in Europe [28,29,32,35,48,49,52,54,55,58]. For most instruments (n=20, 61%), no theoretical basis for the development of the instruments was reported (Table 2) [29,31,35,37,39-41,43,45-48,50,52-58]. The Lily Model served as the theoretical basis for 8 (24%) instruments [6,30,32,34,36,42,44,49]. The remaining instruments not developed from scratch without any theoretical foundation were based on other theories, such as the Transactional Model of eHealth Literacy (TMeHL) [20].

Across all instruments, a total of 209 original dimensions of DHL were identified (Multimedia Appendix 1).

The number of original dimensions per instrument ranged from 2 [33,43] to 22 [53]. The sample size of the studies on instrument development ranged from 15 [29] to 1914 participants [34]. Of these instruments, 12 (36%) were designed for the general population [6,28,30,31,37,47,48,51,54,55,57,58], and 21 (64%) targeted specific population groups [20,29,32-36,38-46,49,50,52,53,56], such as students or older people with chronic diseases. The average age of participants in these studies ranged from 12.9 (SD 0.9) [42] to 71.27 (SD 10.82) years [45]. In terms of gender, 14 (43%) studies had an almost equal proportion of male and female participants [20,30-34,36,44-46,51,54,55,57,58]. In the remaining studies, the study population was predominantly women (n=12, 36%) [29,35,37,40-42,47,49,50,53,56] and men (n=4, 12%) [6,43,52,54]. Three (9%) studies [28,39,48] did not report on the gender distribution in the analyzed population.

The DHLMIs varied in their design: 7 (21%) of the instruments measured DHL on a performance basis [29,37,38,40,47,53,55], 20 (61%) applied self-reported measurements [6,20,28,30-33,35,39,42-46,48-52,57], and 6 (18%) combined both approaches [29,34,36,41,54,56].

**Table 2.** Characteristics of included measurement instruments.

Instrument(s)	Author, year	Theory	Country	Participants included in primary studies, N	Generic instrument or for specific target group	Age (y), mean (SD) or range	Women (%)	Performance based or self-reported
CeHLS-D^a^	Lee et al, 2022 [43]	None specified	South Korea	453	T^b^ (patients with chronic diseases)	56.8 (10.8)	160 (35.3)	S^c^
DHLA^d^ (adapted version)	Liu et al, 2020 [47]	None specified	Taiwan	1871	G^e^	NA^f^	1011 (63.7)	P^g^
DHLAT^h^ (adapted version)	St. Jean et al, 2017 [53]	None specified	United States	19	T (young people aged 12-15 y)	12.9 (0.9)	12 (63.2)	P
DHLC^i^	Rachmani et al, 2022 [51]	DigComp 2.0	Indonesia	383	G	37.59 (12.69)	219 (57.2)	S
DHLI^j^	van der Vaart and Drossaert, 2017 [58]	None specified	The Netherlands	200	G	46.4 (19.0)	107 (53.5)	S and P
DHLS^k^	Nelson et al, 2022 [50]	None specified	United States	508	T (caregivers of young children)	34.7 (7.7)	454 (89.4)	S
DHRQ^l^	Scherrenberg et al, 2023 [52]	None specified	Belgium	315	T (patients in a clinical context)	62.6 (15.1)	118 (37.5)	S
DHTL-AQ^m^	Yoon et al, 2022 [33]	Health behavior model and unified theory of acceptance use of technology	South Korea	590	T (adults who use digital health technologies)	46.5 (13.0)	317 (53.7)	S
eHealth Literacy Scale 2.0	Li, 2018 [44]	Lily Model and social cognitive theory (self-efficacy theory)	United States	238	T (low income and socially marginalized population)	47.1 (13.63)	175 (75.10)	S
eHEALS^n^	Norman and Skinner, 2006 [6]	Lily Model and social cognitive theory (self-efficacy theory)	Canada	664	G	14.95 (1.24)	294 (44)	S
eHEALS+not specified	Neter and Brainin, 2017 [34]	Lily Model	Israel	82	T (adults who use the internet for health-related purposes)	67 (11)	49 (60)	S and P
eHEALS+not specified	van der Vaart et al, 2011 [54]	None specified	The Netherlands	189 and 88	G	52 (11)^o^ and 43 (18)^p^	70 (37)^o^ and 43 (49)^p^	S and P
eHEALS+not specified	Xie, 2011 [56]	None specified	United States	124	T (older adults)	68.15 (9.00)	82 (66.1)	S and P
eHEALS-E^q^	Petric et al, 2017 [32]	Lily Model and social cognitive theory (self-efficacy theory)	Slovenia	644	T (users of online health communities)	40	535 (83)	S
eHEALS-6	Lin et al, 2021 [45]	None specified	China	215	T (older adults)	71.27 (10.82)	122 (56.7)	S
eHLA^r^	Karnoe et al, 2018 [30]	Lily Model and eHLF^s^	United States	475	G	NR^t^	245 (51.6)	S
eHLQ^u^	Kayser et al, 2018 [28]	eHLF	Denmark	475	G	NR	—^v^	S
eHLS^w^	Hsu et al, 2014 [39]	None specified	Taiwan	525	T (college students)	NR	—	S
e-HLS^x^	Seçkin et al, 2016 [31]	None specified	United States	710	G	48.82 (16.43)	381 (53.7)	S
eHLS-Web 3.0^y^	Liu et al, 2021 [46]	None specified	China	421	T (college students)	20.5 (1.4)	218 (51.8)	S
eHLT^z^+eHLR^aa^	Camiling, 2019 [36]	Lily Model	Philippines	274	T (high school students)	NR	146 (53.3)	S/P
GR-eHEALS^ab^	Marsall et al, 2022 [49]	Lily Model and social cognitive theory (self-efficacy theory)	Germany	470	T (German adult population)	37.16 (13.4)	332 (70.6)	S
HL19-DIGI^ac^	M-POHL, 2021 [48]	None specified	Austria, Belgium, Czech Republic, Denmark, France, Germany, Hungary, Ireland, Israel, Norway, Portugal, Slovakia, and Switzerland	NA	G	NA	—	S
PB-mHLS^ad^	Zhang and Li, 2022 [57]	None specified	China	552	G	NR	264 (47.8)	S
PRE-HIT^ae^	Koopman et al, 2014 [42]	Lily Model	United States	200	T (older patients with chronic diseases)	54	142 (71)	S
READHY^af^	Kayser et al, 2019 [35]	None specified	Denmark	305	T (patients with chronic diseases)	58	216 ( 70.8)	S
RRSA^ag^	Ivanitskaya et al. 2006 [41]	None specified	United States	308	T (college students)	18‐23	237 (77)	S/P
RRSA (adapted version)	Ivanitskaya et al, 2010 [40]	None specified	United States	1914	T (college students)	NR	1397 (73)	P
RRSA-h^ah^	Hanik and Stellefson, 2011 [38]	Two-process theory of human information processing	United States	77	T (undergraduate health education students)	21.3 (2.0)	68 (88.3)	P
TeHLI^ai^	Paige et al, 2019 [20]	TMeHL^aj^	United States	283	T (older people)	64.34 (10.49)	160 (56.5)	S
Not specified	Chan and Kaufman, 2011 [37]	None specified	United States	20	G	18‐65	14 (70)	P
Not specified [29]	van der Vaart et al, 2013 [29]	None specified	The Netherlands	15^o^ and 16^p^	T (patients with rheumatic diseases)	56.4 (10.5)^o^ and 48.6 (14.2)^p^	12 (80)^o^ and 13 (81)^p^	P
Not specified	van Deursen and van Dijk, 2011 [55]	None specified	The Netherlands	88	G	18‐80	43 (49)	P

a CeHLS-D: Condition-specific eHealth literacy Scale for Diabetes.

b T: specific target group.

c S: self-reported.

d DHLA: digital health literacy assessment.

e G: generic.

f Not available.

g P: perfomanced based.

h DHLAT: digital health literacy assessment tool.

i DHLC: digital health literacy competencies for citizens.

j DHLI: digital health literacy instrument.

k DHLS: Digital Health Care Literacy Scale.

l DHRQ: Digital Health Readiness Questionnaire.

m DHTL-AQ: Digital Health Technology Literacy Assessment Questionnaire.

n eHEALS: 8-item eHealth Literacy Scale.

o Study 1.

p Study 2.

q eHEALS-E: eHealth Literacy Scale–extended.

r eHLA: eHealth literacy assessment toolkit.

s eHLF: eHealth literacy framework.

t NR: not reported.

u eHLQ: eHealth Literacy Questionnaire.

v Not applicable.

w eHLS: eHealth Literacy Scale.

x e-HLS: Electronic Health Literacy Scale.

y eHLS-Web 3.0: eHealth Literacy Scale Web 3.0.

z eHLT: eHealth Literacy Test.

aa eHLR: eHealth Literacy Rubric.

ab GR-eHEALS: German eHealth Literacy Scale.

ac HL19-DIGI: Health Literacy Survey 19 Digital.

ad PB-mHLS: problem-based mHealth Literacy Scale.

ae PRE-HIT: The Patient Readiness to Engage in Health Internet Technology instrument.

af READHY: Readiness and Enablement Index for Health Technology.

ag RRSA: Research Readiness Self-Assessment.

ah RRSA-h: Research Readiness Self-Assessment-health scale.

ai TeHLI: transactional eHealth literacy instrument.

aj TMeHL: transactional model of eHealth literacy.

**Table 3.** Details of instruments measuring digital health literacy included in the reviews and their frequency of use.

Review (author, year)	DHLMIs^a^ and frequency of their use in primary studies											
	eHEALS^b^ [6] (n=163)	eHLQ^c^ [28] (n=13)	DHLI^d^ [58] (n=12)	eHLA^e^ [30] (n=8)	TeHLI^f^ [20](n=5)	e-HLS^g^ [31](n=3)	DHTL-AQ^h^ [33] (n=2)	eHEALS+not specified [34] (n=2)	eHEALS-E [32](n=3)	READHY^i^ [35] (n=2)	Other [29,36-57] (n=21)	Total
Ahn and Kim, 2024 [4]	3	1	1	1	1	1	1		1		7	17
Bai et al, 2025 [22]	57	2	5						1		2	67
Crocker et al, 2023 [16]				3				1			10	14
Délétroz et al, 2026 [14]	X^1j^	X^1^	X^1^	X^1^	X^1^	X^1^	X^1^		X^1^		X^1^	
Dijkman et al, 2023 [23]	12	4	1	1						1		19
Faux-Nightingale et al, 2022 [11]	14		1	1	1							17
Huang et al, 2023 [24]					1			1				2
Kaihlanen et al, 2023 [25]	8	3	1	1	1	1	1			1	2	19
Lee et al, 2021 [12]	48	2	2	1	1	1			1			56
Wang and Luan, 2022 [26]	X^1^	X^1^	X^1^			X^1^						
Wang et al, 2025 [15]	X^1^	X^1^	X^1^	X^1^	X^1^	X^1^	X^1^		X^1^		X^1^	
Xie et al, 2022 [27]	21	1	1									23

a DHLMI: digital health measurement instrument.

b eHEALS: 8-item eHealth literacy scale.

c eHLQ: eHealth literacy questionnaire.

d DHLI: digital health literacy instrument.

e eHLA: eHealth Literacy Assessment toolkit.

f TeHLI: Transactional eHealth Literacy Instrument.

g e-HLS: Electronic Health Literacy Scale.

h DHTL-AQ: Digital Health Technology Literacy Assessment Questionnaire.

i READHY: Readiness and Enablement Index for Health Technology.

j X1: no information on the frequency of the measurement instrument in primary studies.

### Overview of Assigned Dimensions

**Figure 2.**
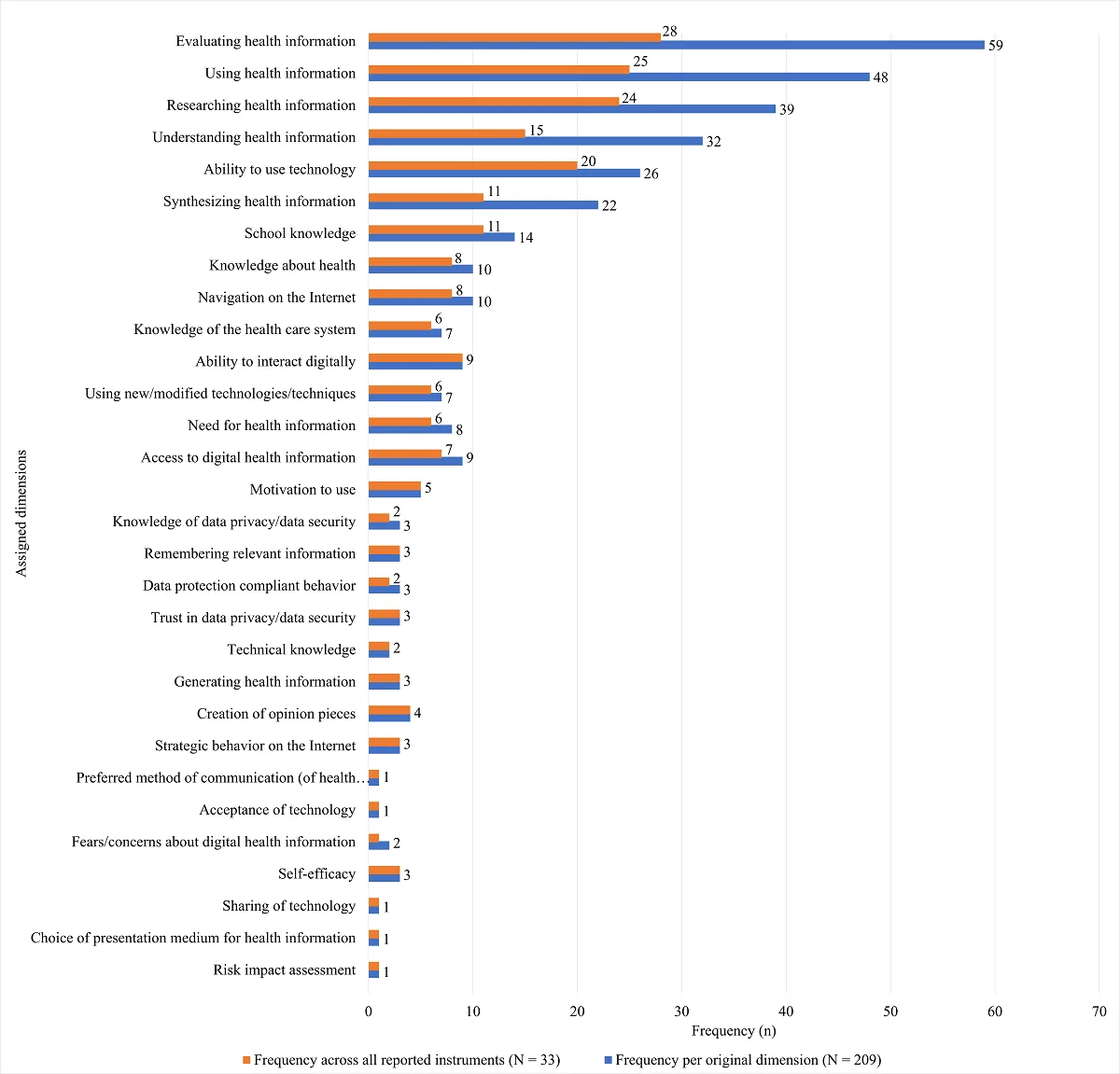
Frequency of use of the assigned dimensions.

The number of assigned dimensions per instrument and per original dimension is shown in Figure 2.

A total of 30 dimensions were assigned during the qualitative content analysis. For some dimensions, a clear categorization was not possible; this occurred in 17 cases for the frequency per original dimension and in 7 cases across all reported instruments. The most frequently assigned dimension was “evaluating health information.” This dimension was measured in 28 (84%) of the 33 instruments. This was followed by “using health information” (n=25, 76%), “researching health information” (n=24, 73%), “ability to use technology” (n=20, 61%), and “understanding health information” (n=15, 45%). A very similar order emerged when counting the assigned dimensions according to the original dimensions of the measurement instruments: the dimension “evaluation of health information” was measured most frequently, accounting for 59 of the 209 original dimensions. Consequently, this dimension was operationalized in 59 different ways (“approaches”). The other most commonly reported dimensions were “using health information” (n=48, 23%), “researching health information” (n=39, 19%), “understanding health information” (n=32, 15%), and “ability to use technology” (n=26, 12%).

**Figure 3.**
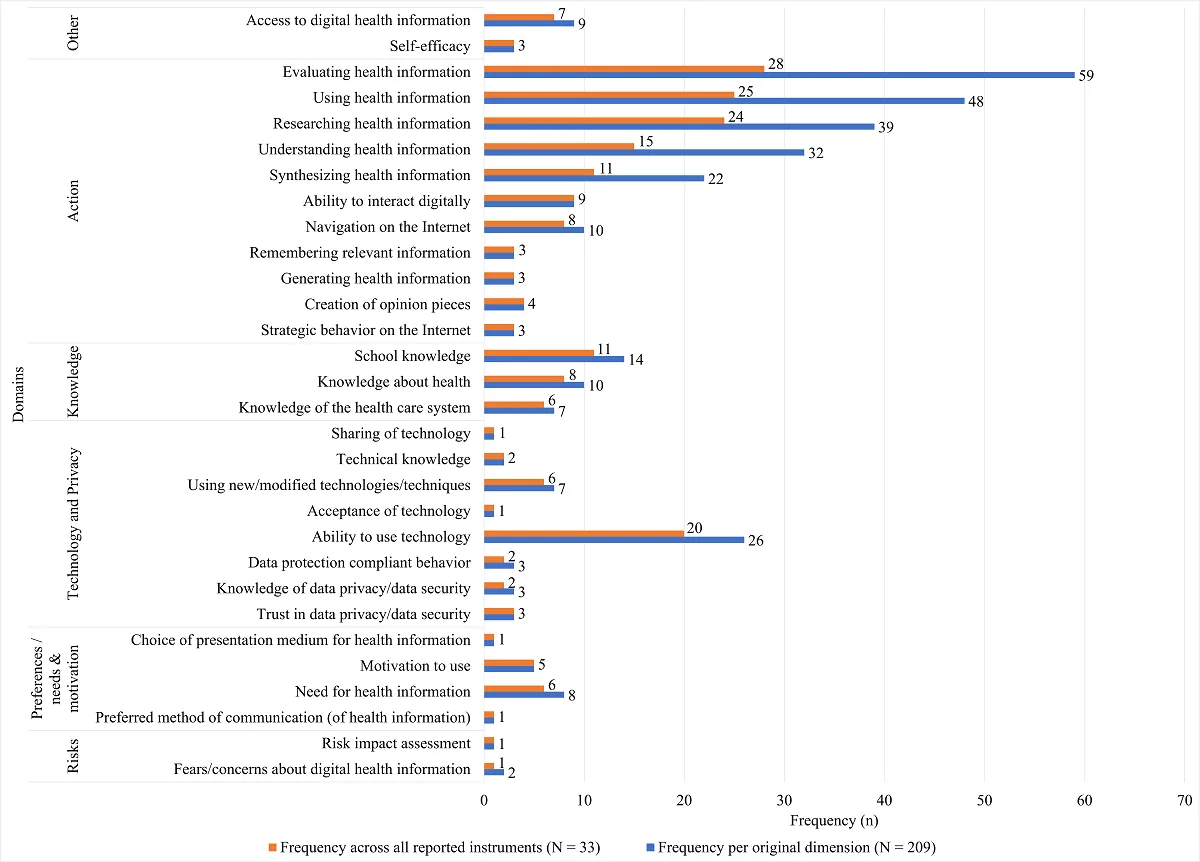
Frequency of use of the assigned dimensions by domains. Action includes skills like understanding, using, and evaluating health information, as well as digital interaction and navigation. Knowledge refers to factual knowledge such as understanding the healthcare system, school-taught content, and general health knowledge. Preferences/needs & motivation encompasses preferred formats, need for health information, and motivation to engage with it. Risks from health information involves concerns and perceived risks related to digital health information. Technology & Privacy covers data protection, trust in digital security, technology use and sharing, and technical knowledge.

The assigned dimensions derived from the original dimensions could be sorted into 6 domains (Figure 3): “Action,” “Knowledge,” “Technology & Privacy,” “Preferences/Needs & Motivation,” “Risks From Health Information,” and “Other.” Four of the 5 most used categories (see above) belong to the “Action” domain. The dimensions from the domain “Risks from Health Information” were used least frequently. The domains covered by each instrument are presented in Table 4. A codebook containing the domains, their assigned dimensions, and descriptions is provided in Multimedia Appendix 2.

**Table 4.** Categories (domains) of the digital health literacy assessment tools.

Instruments	Author, year	Action	Technology and privacy	Knowledge	Preferences or needs and motivation	Risks from health information	Categorization not possible	Other
CeHLS-D^a^	Lee et al, 2022 [43]	X	X					
DHLA^b^ (adapted version)	Liu et al, 2020 [47]	X		X			X	
DHLAT^c^ (adapted version)	St. Jean et al, 2017 [53]	X		X				
DHLC^d^	Rachmani et al, 2022 [51]	X	X	X				
DHLI^e^	van der Vaart and Drossaert 2017 [58]	X	X					
DHLS^f^	Nelson et al, 2022 [50]						X	
DHRQ^g^	Scherrenberg et al, 2023 [52]	X	X		X			
DHTL-AQ^h^	Yoon et al, 2022 [33]	X	X					
eHealth Literacy Scale 2.0	Li, 2018 [44]	X	X					X
eHEALS^i^	Norman and Skinner, 2006 [6]	X	X	X				X
eHEALS+not specified	Neter and Brainin, 2017 [34]	X	X	X			X	X
eHEALS+not specified	van der Vaart et al, 2011 [54]	X	X	X				X
eHEALS+not specified	Xie, 2011 [56]	X	X	X				X
eHEALS-E^j^	Petric et al, 2017 [32]	X			X			
eHEALS-6	Lin et al, 2021 [45]	X			X			X
eHLA^k^	Karnoe et al, 2018 [30]	X	X	X	X			
eHLQ^l^	Kayser et al, 2018 [28]	X	X	X	X			
eHLS^m^	Hsu et al, 2014 [39]	X		X				
e-HLS^n^	Seçkin et al, 2016[31]	X						
eHLS-Web 3.0^o^	Liu et al, 2021 [46]	X						
eHLT^p^+eHLR^q^	Camiling, 2019 [36]	X	X					
GR-eHEALS^r^	Marsall et al, 2022 [49]	X	X	X	X	X		X
HL19-DIGI^s^	M-POHL, 2021 [48]	X	X					
PB-mHLS^t^	Zhang and Li, 2022 [57]	X	X	X	X			X
PRE-HIT^u^	Koopman et al, 2014 [42]		X		X	X	X	
READHY^v^	Kayser et al, 2019 [35]	X	X	X			X	X
RRSA	Ivanitskaya et al, 2006 [41]	X	X	X			X	
RRSA^w^ (adapted version)	Ivanitskaya et al, 2010 [40]	X	X	X			X	
RRSA-h^x^	Hanik and Stellefson 2011 [38]	X						
TeHLI^y^	Paige et al, 2019 [20]	X	X			X		
Not specified	Chan and Kaufman, 2011 [37]	X	X	X	X			
Not specified	van der Vaart et al, 2013 [29]	X	X					
Not specified	van Deursen et al, 2011 [55]	X	X					

a CeHLS-D: Condition-specific eHealth literacy Scale for Diabetes.

b DHLA: digital health literacy assessment.

c DHLAT: digital health literacy assessment tool

d DHLC: digital health literacy competencies for citizens.

e DHLI: digital health literacy instrument.

f DHLS: Digital Health Care Literacy Scale.

g DHRQ: Digital Health Readiness Questionnaire.

h DHTL-AQ: Digital Health Technology Literacy Assessment Questionnaire.

i eHEALS: 8-item eHealth Literacy Scale.

j eHEALS-E: eHealth Literacy Scale-Extended.

k eHLA: eHealth Literacy Assessment Toolkit.

l eHLQ: eHealth Literacy Questionnaire.

m eHLS: eHealth Literacy Scale.

n e-HLS: Electronic Health Literacy Scale.

o eHLS-Web 3.0: eHealth Literacy Scale Web 3.0.

p eHLT: eHealth Literacy Test.

q eHLR: eHealth Literacy Rubric.

r GR-eHEALS: German eHealth Literacy Scale.

s HL19-DIGI: Health Literacy Survey 19 Digital.

t PB-mHLS: Problem-Based mHealth Literacy Scale.

u PRE-HIT: The Patient Readiness to Engage in Health Internet Technology Instrument.

v READHY: Readiness and Enablement Index for Health Technology.

w RRSA: Research Readiness Self-Assessment.

x RRSA-h: Research Readiness Self-Assessment-Health Scale.

y TeHLI: transactional eHealth literacy instrument.

## Discussion

### Principal Findings

Our analysis identified 33 DHLMIs from 12 reviews. Almost two-thirds (n=20, 61%) of these instruments were not based on a theory. The majority of the instruments (21/33, 64%) were developed for a specific target group. Most of them (20/33, 61%) assessed DHL via self-report, while only 21% (7/33) relied exclusively on performance-based assessment.

The analysis of the measured dimensions revealed a wide variety of DHL dimensions across the instruments. However, the qualitative content analysis revealed that there are key dimensions of DHL that are captured in almost all instruments, but often under different names. These key dimensions include evaluating health information, using health information, researching health information, and the ability to use technology. Such key dimensions covered by a majority of instruments were defined and operationalized differently throughout the included studies and instruments, severely limiting the comparability of results [59]. This problem could be due to a low discriminant validity of health literacy subconstructs [60], of which DHL is one, with navigational health literacy being another, which partly covers similar constructs [61].

The lack of any theory as a basis for several of the measurement instruments analyzed is especially noteworthy because existing models are based on existing evidence and definitions themselves [7]. Thus, the use of acknowledged models for instrument development ensures content validity [62]. Furthermore, comparability of results of different instruments is more feasible when they are based on the same theoretically sound dimensions [63]. That being said, the wide number of assigned dimensions within our content analysis that applied to more than one original dimension, and, likewise, the number of original dimensions that fit into more than one assigned dimension hint at a limited comparability of the results the measurement instruments we studied will likely gain [59].

Comparability of results, however, is the basis for the generalization of study results and distinguishing between target groups is only possible based on a unified set of dimensions on which said groups might differ [64]. It is because of considerations such as this that researchers call for core outcome sets in the measurement of key dimensions to be measured in evaluation studies [65], not only, but also concerning digital health applications.

We acknowledge that DHL, such as health literacy, is a context-dependent, dynamic construct, with the required competencies varying substantially across societies, health care systems, and target populations [66-68]. This contextual dependency, combined with the diversity of target populations (eg, older adults vs students), application contexts (eg, clinical vs community settings), and assessment methods (eg, self-reported vs performance-based measures), makes developing a single, universal DHLMI a significant challenge. Nevertheless, this does not preclude identifying a common conceptual core. A Core Outcome Set of key DHL dimensions would not prescribe a fixed, universal instrument; rather, it would provide a shared dimensional framework that can be adapted to specific cultural contexts, health care systems, and target populations. This approach would enable researchers to use contextually appropriate instruments while ensuring that a common set of dimensions is covered. This would increase the comparability of findings across studies that use different DHLMIs.

Our qualitative content analysis of DHL dimensions builds on and extends the work of Wang et al [15], who also conducted a content analysis of eHL instrument dimensions. However, the 2 analyses differ in scope due to differences in inclusion criteria, which likely explain differences in the resulting dimension landscape.

Wang et al restricted inclusion to studies whose primary focus was the development of a new DHL instrument, explicitly excluding studies in which instrument development was not the main objective. Furthermore, they included only instruments developed for the general population, excluding instruments designed for specific target groups. Our review, in contrast, included both instrument development studies and application studies—for example, studies evaluating digital health interventions or examining DHL as an outcome in clinical or community settings—as well as instruments developed for specific populations, such as older adults or patients with chronic conditions. This broader inclusion allows our analysis to reflect more closely which instruments and dimensions are actually used in research practice, rather than being limited to the subset of instruments and dimensions prominent in the instrument development literature.

As a result, our analysis captures a broader and more heterogeneous range of dimensions, which may partly be explained by the varying constructs and comparison measures used during instrument development and validation. Instruments were often validated against different related concepts, including health literacy, digital literacy, self-efficacy, or technology use, reflecting divergent assumptions regarding the conceptual boundaries of DHL. This may contribute to the considerable heterogeneity observed across existing measurement tools.

Both our analysis and that of Wang et al confirmed the observation that discrepancies in the conceptualization of DHL across instruments limit comparability—a challenge that becomes even more apparent when the full breadth of instruments in active research use is considered. Comparability and synthesis of research findings are essential to provide informed and comprehensive insights into the current state of DHL. Such insights form the foundation for the development of targeted policies and interventions aimed at strengthening DHL at national and international levels. To date, assessments of DHL have relied primarily on separate primary data collections, such as the national survey conducted in Germany [69]. A more efficient and systematic approach would involve aggregating findings from existing studies through meta-analyses, which allow for a structured integration and comparison of results across contexts and populations, or even across regions and countries. Standardizing measurement instruments would also improve comparability and enable earlier identification of trends, gaps in health care, and emerging needs, which are critical elements for evidence-based policy-making [70].

For the effective implementation of digital health care solutions, it would be both desirable and necessary—although still rarely practiced—for health care providers to assess patients’ DHL before suggesting or prescribing the use of such solutions [71]. This consideration motivated our initial intention to provide a short-form measurement instrument suitable for daily health care practice and its time restraints [23]. Systematic assessment would help ensure that digital solutions reach those who can benefit most, thereby improving both individual health outcomes and quality of care while at the same time reducing health disparities due to a digital divide [23].

The large number of measurement instruments available also poses a challenge for developers of digital health solutions, making it difficult to identify and select appropriate tools for assessing the impact of such interventions on DHL [72]. This issue is particularly relevant in the context of effectiveness assessments, such as those required for approval of the German digital health application. The selection of the most appropriate and meaningful instrument to demonstrate effectiveness remains an open question in this process [73]. Furthermore, the development of digital interventions specifically designed to improve, rather than merely assess, DHL requires a solid theory and validated measurement tools that capture the core dimensions of DHL [74].

### Strengths and Limitations

To the best of our knowledge, this is the first review to synthesize both self-reported and performance-based instruments for measuring DHL with a structured qualitative content analysis to systematically analyze and synthesize the dimensions covered by DHLMIs across both instruments developed for the general population and those targeting specific populations. We also provide an overview of available instruments, categorized by target group and a theory. This overview offers a foundation for the refinement of existing or the development of new instruments and serves as practical guidance for researchers, practitioners, and policymakers when selecting suitable tools.

A particular strength of this review is its inclusive conceptual scope. Reflecting the conceptual heterogeneity of DHL, our review systematically mapped all available instruments, not restricting inclusion to those operationalizing health information competencies alone. The resulting dimensional framework therefore extends beyond information-seeking and appraisal to encompass competencies such as technology use, interactive engagement, and the ability to actively generate and share health-related content. These dimensions are directly relevant to the use of digital health services. This broad coverage means that the findings of this review are relevant not only to health information contexts but also to the growing landscape of digital health service use.

However, our review also has limitations. It is neither a full-scale systematic nor an umbrella review [75]. Despite our narrative approach, we have applied several systematic principles in key areas of our work. These include defining a clear research question, applying explicit inclusion and exclusion criteria, describing the selection process for reviews and instruments, conducting structured data extraction, and transparently presenting and discussing the results. Given the systematic nature of our approach in many respects, we considered using the term “review of reviews” but decided against it because our work does not meet all the associated criteria and we did not want to misrepresent our methodology. The chosen narrative format nevertheless seems appropriate, as the research field is conceptually heterogeneous and the included studies vary considerably in methodology and research focus. It is precisely this heterogeneity that precluded a quantitative synthesis of the findings. It should also be noted that the theoretical framework of DHL remains incomplete. Existing theoretical frameworks, including those underlying the 8-item eHealth Literacy Scale and related eHealth competency models, do not yet provide a sufficient basis for the creation of a comprehensive model that covers the many dimensions of this field. When theory is insufficient to capture the complexity of a phenomenon, qualitative and interpretive methods can support concept development. Another limitation is that only measurement instruments that have already been analyzed in a review were taken into account. Therefore, very new measurement instruments, for example, were not included in our analysis. As our study focused on the dimensions of DHL rather than the quality of the measurement instruments, it cannot make any statements about their validity or other quality characteristics. Furthermore, future studies should examine the measuring instruments’ test quality criteria to establish a solid basis for further research.

Furthermore, we did not extract data on translated, cross-culturally adapted, or validated versions of the included instruments, as this was not one of the objectives specified in advance for our review. Future reviews of instruments for measuring DHL could systematically document the available language versions and cross-cultural validation studies. This would enable a more comprehensive assessment of the international applicability and dissemination of the instruments and the dimensions of DHL they measure.

Qualitative content analysis was not done independently; however, category formation was done by 2 researchers in open discussion and checked by a third.

When considering the “key dimensions” identified through qualitative content analysis, it is important to acknowledge that they are based on measurement instruments whose validity should be critically examined—particularly those relying solely on self-report [12]. Therefore, while these key dimensions should be interpreted with caution, they still form part of the current evidence base. 

Our qualitative approach to analyze the existing instruments allowed us to uncover underlying dimensions of DHL that should be covered by a consolidated instrument in the future. From a purely scientific standpoint, our work underlines the need for instruments that are based on theory as well. Future work on a Core Outcome Set for the evaluation of digital health applications will therefore profit from our work as well.

### Conclusions

Our review has 3 direct implications for research and practice. First, researchers can use the resulting dimension profiles to make more informed and transparent decisions when selecting an instrument suited to their research question and target population. Second, instrument developers gain an evidence-based starting point for the conceptual grounding of new or revised instruments. Third, the identified dimensional core across instruments provides a foundation for the development of a core outcome set for DHL, which could support greater comparability across studies in the future.

Given the contextual dependency of DHL across different societies and health care systems, the goal is not to develop a single universal instrument but to establish a shared conceptual core of key dimensions that can serve as a basis for culturally and population-specific adapted instruments, ultimately supporting greater comparability of findings across diverse research contexts.

## Supplementary material

10.2196/91603 Multimedia Appendix 1 Dimensions of digital health literacy of included measurement instruments.

10.2196/91603 Multimedia Appendix 2 Codebook for domains, assigned dimensions, and descriptions.

## Abbreviations

DHL: digital health literacy
DHLMI: digital health measurement instrument
DiGA: digital health applications
TMeHL: Transactional Model of eHealth Literacy
