# Supplementary material for: How Many Instruments Do We Need? Strengths, Weaknesses, and Potentials for Further Developments of Digital Health Literacy Measurement Instruments: Narrative Overview of Reviews and Content Analysis
Source: Interact J Med Res. 2026 Aug 28;15:e91603. doi: 10.2196/91603 (PMC13524364; doi:10.2196/91603)
Supplement: Supplementary file 1 [file ijmr-v15-e91603-s001.docx]

| Instrument(s) | Author, year | Original dimensions of digital health literacy |
| --- | --- | --- |
| CeHLS-D {Lee, 2022 #106} | Lee et al. 2022 | - cognitive Actions for Internet Diabetes Information - abilities of Digital Communication |
| DHLA (adapted version) {Liu, 2020 #108} | Liu et al. 2020 | - traditional literacy (eHEALS) - computer literacy (eHEALS) - information literacy (eHEALS) - health literacy (eHEALS) - media literacy (eHEALS) - science literacy (eHEALS) - trust in health information from folklore and customs - ability to use information technology |
| DHLAT (adapted version) {St. Jean, 2017 #122} | St. Jean et al. 2017 | - awareness of search engines and their capabilities - understand how search engines work - develop and refine a range of questions to frame search - understand relevant health terms - develop appropriate search strategies - use correct keyword searching strategies - use correct spelling in search terms - evaluate source (e.g., sponsor or type of site) to determine the believability of the person - providing the information - reduce search result selection based solely on word familiarity - aware of primary health resources to begin search - access valid information, products, and services - use the library’s electronic resources, such as databases, etc. - evaluate information based on its accuracy, validity, and appropriateness - evaluate the credibility of the medium - evaluate the site based on when it was last updated - limit reliance on surface characteristics, such as the design of a website, the language used, etc. - conclude which sites/information are valid and accurate by using conscious strategies (rather than simply using intuitive and heuristic judgments) - make sense of information gathered from diverse sources by identifying misconceptions, main and supporting ideas, conflicting information, point of view, and bias - organize gathered information to optimize future retrieval/use - synthesize information from multiple sources; draw conclusions - open-ended, so participant may have touched on any of the literacy bits we identified in our earlier work |
| DHLC {Rachmani, 2022 #118} | Rachmani et al. 2022 | - information and data Literacy - communication and collaboration - digital content creation - safety - problem solving |
| DHLI {van der Vaart, 2017 #125} | van der Vaart & Drossaert 2017 | - operational skills - navigation skills - information search skills - adding self-created content to web-based applications - protecting and respecting privacy when using the internet - evaluating information |
| DHLS {Nelson, 2022 #112} | Nelson et al. 2022 | - confidence in using applications/ programs - ability to set up video chats - ability to solve or troubleshoot basic technical issues |
| DHRQ {Scherrenberg, 2023 #119} | Scherrenberg et al. 2023 | - digital usage - digital skills - digital literacy - digital health literacy - digital learnability |
| DHTL-AQ {Yoon, 2022 #132} | Yoon et al. 2022 | - digital functional literacy - digital critical literacy |
| eHealth Literacy Scale 2.0 {Li, 2018 #232} | Li et al. 2018 | - NA |
| eHEALS {Norman, 2006 #114} | Norman & Skinner 2006 | - traditional literacy - computer literacy - information literacy - health literacy - media literacy - science literacy |
| eHEALS + not specified {Neter, 2017 #113} | Neter and Brainin 2017 | - traditional literacy (eHEALS) - computer literacy (eHEALS) - information literacy (eHEALS) - health literacy (eHEALS) - media literacy (eHEALS) - science literacy (eHEALS) - accessing information - understanding information - appraising information - applying information - generating information - operational skills - formal skills - information skills - strategic skills |
| eHEALS + not specified {van der Vaart, 2011 #127} | van der Vaart et al. 2011 | - traditional literacy (eHEALS) - computer literacy (eHEALS) - information literacy (eHEALS) - health literacy (eHEALS) - media literacy (eHEALS) - science literacy (eHEALS) - operational internet skills - formal internet skills - information internet skills - strategic internet skills |
| eHEALS + not specified {Xie, 2011 #131} | Xie et al. 2011 | - traditional literacy (eHEALS) - computer literacy (eHEALS) - information literacy (eHEALS) - health literacy (eHEALS) - media literacy (eHEALS) - science literacy (eHEALS) - perceived usefulness of the - internet in helping make health decisions - perceived importance of being able to access health resources on the - Internet - evaluation the quality of health websites |
| eHEALS-E {Petrič, 2017 #116} | Petric et al. 2017 | - traditional literacy (eHEALS) - computer literacy (eHEALS) - information literacy (eHEALS) - health literacy (eHEALS) - media literacy (eHEALS) - science literacy (eHEALS) - awareness of sources - recognising quality and meaning - understanding information - perceived efficiency - maintaining a critical awareness of biases in validating information - being smart on the Net |
| eHEALS-6 {Lin, 2021 #224} | Lin et al. 2021 | - locating health information - retrieving health information - evaluating health information - applying health information |
| eHLA {Karnoe, 2018 #101} | Karnoe et al. 2018 | - functional health literacy - self-assessed health literacy - familiarity with health and health care - knowledge of health and disease - familiarity with technology - technology confidence - incentives for engaging with technology |
| eHLQ {Kayser, 2018 #102} | Kayser et al. 2018 | - using technology to process health information - understanding of health concepts and language - ability to actively engage with digital services - feel safe and in control - motivated to engage with digital services - access to digital services that work digital services that suit individual needs |
| eHLS {Hsu, 2014 #98} | Hsu et al. 2014 | - functional eHealth Literacy - interactive eHealth Literacy - critical eHealth Literacy |
| e-HLS {Seçkin, 2016 #121} | Seçkin et al. 2016 | - trust - action - behavior |
| eHLS-Web 3.0 {Liu, 2021 #107} | Liu et al. 2021 | - acquisition - verification - application |
| eHLT + eHLR {Camiling, 2019 #90} | Camiling et al. 2019 | - traditional literacy and numeracy - health and information - computer |
| GR-eHEALS {Marsall, 2022 #230} | M-POHL 2021 | - Digital Health Literacy – dealing with digital health information - Interaction with digital resources for health - Use of digital devices for health |
| HL19-DIGI {M-POHL., 2021 #237} | Marshall et al. 2022 | - traditional literacy (eHEALS) - computer literacy (eHEALS) - information literacy (eHEALS) - health literacy (eHEALS) - media literacy (eHEALS) - science literacy (eHEALS) - NA |
| PB-mHLS {Zhang, 2022 #133} | Zhang et al. 2022 | - mHealth desire - mobile phone operational skills - acquiring mHealth information - acquiring mHealth services - understanding of medical terms - mobile-based patient-doctor communication - evaluating mHealth information - mHealth decision-making |
| PRE-HIT {Koopman, 2014 #104} | Koopman et al. 2014 | - health information need - computer/Internet experience - computer anxiety - preferred mode of interaction - relationship with doctor - cell phone expertise - Internet privacy concerns - no news is good news |
| READHY {Kayser, 2019 #103} | Kayser et al. 2019 | - feel understood and supported by healthcare providers - digital services that suit individual needs - access to digital services that work - motivated to engage with digital services - feel safe and in control - social support for health - ability to actively engage with digital services - understanding of health concepts and language |
| RRSA {Ivanitskaya, 2006 #100} | Ivanitskaya et al. 2006 | - finding health information - evaluating health information - understanding plagiarism - declarative knowledge - procedural knowledge |
| RRSA (adapted version) {Ivanitskaya, 2010 #99} | Ivanitskaya et al. 2010 | - finding health information - evaluating health information - understanding plagiarism - declarative knowledge - procedural knowledge - internet-related beliefs - Internet behaviors |
| RRSA-h {Hanik, 2011 #94} | Hanik and Stellefson 2011 | - obtaining Electronic Health Information - evaluating Electronic Health Information |
| TeHLI {Paige, 2019 #115} | Paige et al. 2019 | - functional eHealth literacy - communicative literacy - critical literacy - translational literacy |
| Not specified {Chan, 2011 #91} | Chan and Kaufman 2011 | - traditional literacy - computer literacy - information literacy - health literacy - media literacy - science literacy - remembering - understanding - applying - analyzing - evaluating - creating |
| Not specified {van der Vaart, 2013 #126} | van der Vaart et al. 2013 | - operating the computer and Internet browser - navigating and orientating - utilizing search strategies - evaluating relevance and reliability |
| Not specified {van Deursen, 2011 #129} | van Deursen et al. 2011 | - operational Internet skills - formal Internet skills - information Internet skills - strategic Internet skills |
