# Supplementary material for: How Many Instruments Do We Need? Strengths, Weaknesses, and Potentials for Further Developments of Digital Health Literacy Measurement Instruments: Narrative Overview of Reviews and Content Analysis
Source: Interact J Med Res. 2026 Aug 28;15:e91603. doi: 10.2196/91603 (PMC13524364; doi:10.2196/91603)
Supplement: Supplementary file 2 [file ijmr-v15-e91603-s002.docx]

| Domains | Assigned dimensions | Description of the category |
| --- | --- | --- |
| Risks from health information | fears/concerns about digital health information | Concerns that health information from the Internet could increase worries or fears. |
|  | risk impact assessment | Assessing the risks associated with sharing and receiving health information via the internet and social media. |
| Preferences/ needs & motivation | preferred method of communication (of health information) | People's preferences in terms of how they want to receive health information or interact with other people/medical providers, e.g. doctors. |
|  | choice of presentation medium for health information | Ability of individuals to consciously select, interpret and evaluate different presentation media (visual, acoustic, multimedia) for health information and to use them effectively in the respective context. |
|  | need for health information | Individual need to have access to health information, e.g. to understand symptoms, deal with health problems and learn about different medical approaches. |
|  | motivation to use | Motivation to use digital services and technologies, especially in the context of health management or to obtain health information. |
| Technology & Privacy | data protection compliant behaviour | Ability to use digital media securely, responsibly and in compliance with data protection regulations. |
|  | knowledge of data privacy/data security | Knowledge of data protection and security principles in media contexts. |
|  | trust in data privacy/data security | Awareness and the feeling of control over one's own personal data in digital systems. |
|  | acceptance of technology | Positive attitude towards digital technologies, especially with regard to their potential benefits for own health management. |
|  | ability to use technology | Basic digital skills and the ability to use technical devices and digital services effectively. It includes the operation of computers, internet browsers and search engines as well as the secure use of internet-based forms. |
|  | technical knowledge | Knowledge of the possible uses of technical devices and digital services |
| Knowledge | knowledge of the healthcare system | Ability to navigate the healthcare system and basic knowledge of its structure, processes and services. |
|  |  |  |
|  | school knowledge | Basic competences in reading, writing, numerical comprehension and context-specific communication (e.g. in the fields of health, IT or English). |
|  | knowledge about health | Knowledge about the basic functioning of the body, your own current state of health and risk factors. |
| Action | understanding health information | Ability to correctly record health-related information and decipher its meaning. |
|  | generating health information | Ability to actively and independently create, interpret and share health information with others. |
|  |  |  |
|  | creation of opinion pieces | Ability to express yourself consciously and reflectively in online social interactions. |
|  | remembering relevant information | Ability to recall relevant knowledge in order to apply it in current situations. |
|  | using health information | Ability to effectively translate health information into concrete actions and decisions. |
|  | ability to interact digitally | Ability to use digital communication tools effectively to interact, collaborate and adapt to different users and contexts in online multimedia social environments. |
|  |  |  |
|  |  |  |
|  | researching health information | Ability to develop and apply effective search strategies to find reliable, relevant and high quality health information. |
|  | synthesising health information | Ability to bring together information from different sources, recognise their interrelationships and integrate them into their own context. |
|  | evaluating health information | Ability to critically evaluate health information to determine its quality, reliability and relevance. |
|  | strategic behaviour on the Internet | Ability to use the Internet strategically to define personal goals, search for relevant information and apply it in a targeted manner |
|  | navigation on the Internet | Ability to navigate the Internet in an effective and orientated manner, e.g. ability to navigate through different web and menu layouts. |
| Other | self-efficacy | Confidence of a person to make health decisions based on digital health resources and to implement them in their everyday life. |
|  | access to digital health information | Availability and quality of the resources users need to use digital health services. |
| categorisation not possible |  | Neither the definition of the dimension nor the associated items are available or freely accessible. This category is also selected if the definition of the dimensions or categories is not sufficiently described. |
